# HELPING KOREAN AMERICAN OLDER ADULTS THROUGH EDUCATION, RESOURCES, AND VOLUNTEERING: A CASE STUDY OF SOMANG SOCIETY

**DOI:** 10.1093/geroni/igad104.0728

**Published:** 2023-12-21

**Authors:** Jessica Cassidy, Hye-Won Shin, Kathy Lee

**Affiliations:** University of Texas at Arlington, Arlington, Texas, United States; University of California Irvine, Irvine, California, United States; University of Texas at Arlington, Arlington, Texas, United States

## Abstract

Many community organizations serving Korean Americans lack culturally or linguistically tailored services for end-of-life care. Subsequently, Korean American older adults often lack understanding of end-of-life care, particularly advance care planning and hospice. Somang Society is a nonprofit organization serving Korean American older adults and their family caregivers with a mission to promote healthy aging, dignity in later life, and a meaningful death. This study details how Somang Society utilizes education, resources, and volunteer programs to increase the awareness of end-of-life issues among Korean American older adults. The organization provides two distinct community education and volunteer trainings regarding (1) end-of-life care planning program and (2) older adults living with dementia and their family caregivers. They provide community education and volunteer trainings in Korean and expect the volunteers to educate others in the community about the importance of healthy aging, dementia, and advanced care planning. As a result, 15,717 Korean American seniors have filed a living will and advance healthcare directives as of February 2023. In addition, Somang Society emphasizes the importance of whole-body donation for training medical students and healthcare professionals. As a result, 2,134 Korean American seniors have donated or registered for the UCI Willed Body Program through Somang Society since 2008. To further disseminate their programming, the organization actively uses social media to educate Korean Americans on these issues. Their YouTube channel analytics indicate the viewers’ positive responses and the potential impact technology usage can have for sharing education with this hard-to-reach population.

